# Body Mass Index Changes from Before to 3 Years After the COVID-19 Lockdown: A Retrospective Longitudinal Study in a Single Elementary School

**DOI:** 10.3390/children12091157

**Published:** 2025-08-30

**Authors:** Iee Ho Choi, Esther Park, Eun-Jee Lee, Sun-Young Kim, Minsun Kim, Sochung Chung

**Affiliations:** 1 Department of Pediatrics, Jeonbuk National University Children’s Hospital, Jeonju 54896, Republic of Korea; cyiho25@naver.com (I.H.C.); queen4013@hanmail.net (E.P.); 2 Research Institute of Clinical Medicine of Jeonbuk National University-Biomedical Research Institute of Jeonbuk National University Hospital, Jeonju 54907, Republic of Korea; kim4889@hanmail.net; 3 Department of Pediatrics, Jeonbuk National University Medical School, Jeonju 54907, Republic of Korea; 4 College of Nursing, Research Institute of Nursing Science, Jeonbuk National University, Jeonju 54896, Republic of Korea; ejlee@jbnu.ac.kr; 5 Department of Pediatrics, Konkuk University Medical Center, Konkuk University School of Medicine, Seoul 05030, Republic of Korea; scchung@kuh.ac.kr

**Keywords:** body mass index, COVID-19, lockdown, primary school children, weight changes

## Abstract

**Background/Objectives**: The COVID-19 pandemic and related lockdown measures markedly disrupted children’s lives, raising concerns particularly about their weight. We investigated changes in body mass index (BMI) in children by grade and sex, from pre- to post-COVID-19 lockdown, and BMI recovery post-lockdown. **Methods**: We retrospectively reviewed the height, weight, and BMI of students from a single elementary school from 2019 to 2023, excluding 2020 (the year of the COVID-19 lockdown). We conducted longitudinal and cross-sectional studies to examine changes in BMI according to grade and BMI status pre-lockdown (2019) and post-lockdown (2021–2023). **Results:** In the entire student body, the BMI Z-score in 2021, 1 year after the COVID-19 lockdown, had increased significantly compared with that in 2019, 1 year pre-lockdown (*p* = 0.009). However, longitudinal studies in each grade yielded different results. Students who were in the first grade in 2019 experienced a significant decrease in BMI Z-score post-lockdown (*p* = 0.003). In contrast, students who were in other grades showed a significant increase in BMI Z-score post-lockdown, with those who were in third grade in 2019 showing the most significant increase (*p* = 0.027). **Conclusions**: The degree of BMI increase in children and adolescents due to the COVID-19 lockdown was inconsistent. Changes in obesity status post-lockdown varied depending on age and obesity levels pre-COVID-19 lockdown. Therefore, to manage and prevent obesity-related metabolic diseases in the post-COVID-19 era, diverse approaches and strategies tailored to age and obesity grades during the COVID-19 lockdown will need to be adopted.

## 1. Introduction

The COVID-19 pandemic and subsequent lockdown significantly disrupted children’s lives, raising concerns about their health, and particularly their weight [1,2]. Initial predictions pointed toward increased childhood obesity due to reduced physical activity [3,4,5,6], increased screen time [3,4,5,7], and changes in diet [8]. However, deeper investigation revealed a more complex picture, with studies reporting increases, decreases, or no significant changes in body mass index (BMI) across different age groups [9,10,11,12]. This inconsistency emphasizes the need for a better understanding of the multifaceted impact of lockdowns on children’s weight and interacting factors influencing these outcomes.

Beyond the simple equation of caloric intake versus expenditure, the pandemic lockdowns affected elements that impact body composition. While the direct impact of school closures on physical activity is a primary concern [3], the home environment also played a crucial role. Changes in family routines, increased screen time outside of school hours [7], and altered parental feeding practices [13] likely contributed to shifts in children’s lifestyle patterns. The lockdown measures further influenced children’s mental and emotional well-being, leading to potential stress-related eating or decreased motivation for structured physical activity [14]. A recent study highlights the potential for a “long-tail” effect of lockdowns, where disruptions in daily routines and lifestyle persist well beyond the period of strict confinement. It underscores the need to consider these diverse factors when examining the impact of lockdowns on pediatric weight management [15].

We sought to address the conflicting findings on the effect of lockdown during the COVID-19 pandemic on childhood obesity. By providing a more nuanced understanding of the complex relationship between lockdowns and children’s weight, we hoped to inform future interventions to mitigate potential long-term health consequences. Thus, in this study, we investigated BMI changes in elementary school children during and after a period encompassing a COVID-19 lockdown, as compared to before the lockdown. Using a retrospective longitudinal design, we analyzed data from a single school over 3 years, excluding the lockdown year (2020) itself. We examined BMI changes across different grade levels and genders to test the hypothesis that lockdown effects were not uniform across all age groups. Thus, both cross-sectional and longitudinal analyses were conducted to assess the overall impact of the lockdown and to identify age-specific trends in weight changes.

## 2. Materials and Methods

### 2.1. Participants

We collected grade, sex, birthday, age, height, weight, and BMI data of the whole student body of a single elementary school in Jeonbuk, Republic of Korea, from 2019 to 2023, except for 2020, which was the year in which the COVID-19 lockdown was in effect. Our study included the entire student population, rather than a sample. The information of students who had the same birthday, were in the same grade, and were of the same gender was excluded, as this complicated student identification and could lead to errors.

### 2.2. Methods

Height and weight were measured using a digital automatic body measurement device (GAIA KIKO, Jawon Medical, Seoul, Republic of Korea) as part of routine school health checkups. Body mass index (BMI) was calculated as weight in kilograms divided by height in meters squared (kg/m^2^). Height, weight, and BMI are presented as percentiles and Z-scores, which were calculated by age- and sex-specific least mean square method based on the 2017 growth reference values for South Korean children and adolescents developed by the Korean Pediatric Society and Korea Center for Disease Control and Prevention [16]. We classified the students as underweight/normal if they had a BMI Z-score between −2 and 1, as overweight if they had a BMI Z-score between 1 and 1.99, as obese if they had a BMI Z-score between 2 and 2.99, and as severely obese if they had a BMI Z-score over 3.

### 2.3. Statistical Analyses

**Figure 1:**
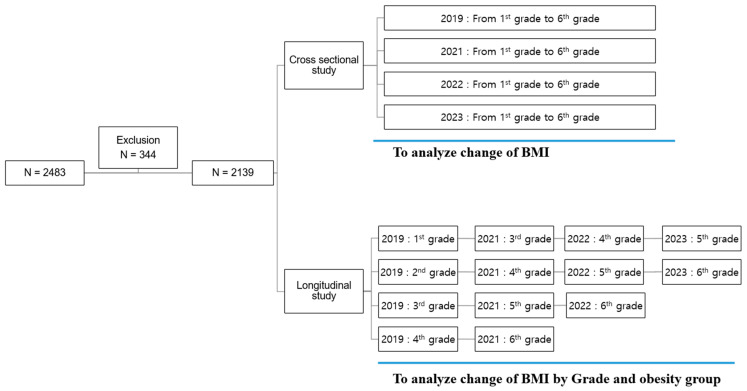
Flow chart of subjects included in this study.

We conducted cross-sectional comparisons of BMI and BMI Z-scores of the whole student body for each year by using the Mann–Whitney test. A longitudinal study was conducted to analyze how the BMI and BMI Z-score of students in each grade, sex, and obesity group changed over the COVID-19 lockdown, using the Wilcoxon signed-rank test (Figure 1). Effect sizes for group comparisons were calculated using Cohen’s d, interpreted as small (0.2), medium (0.5), or large (0.8). The effect size was calculated as the difference between the two means divided by the pooled standard deviation. We used SPSS software version 20 (SPSS, Chicago, IL, USA) to perform all statistical analyses. A *p* value of <0.05 was considered statistically significant.

### 2.4. Ethical Statement

This study was approved by the Institutional Review Board (IRB) of the Clinical Research Institute of Jeonbuk National University Hospital (IRB no. CUH 2025-03-022). The need for obtaining informed participant consent was waived by the IRB due to the retrospective nature of the study.

## 3. Results

### 3.1. Clinical Characteristics of the Study Population

We recruited 2139 individuals for the study, comprising 1108 male individuals and 1031 female individuals. The annual cohort consisted of 541 individuals in 2019 and 2021, 544 in 2022, and 513 in 2023. Table 1 presents the mean and standard deviation of height, weight, BMI, and their respective Z-scores by school grade and gender for each year.

In 2019, 91 students were in first grade, and this cohort had reduced to 90 when they reached third grade in 2021, remained at 90 in fourth grade in 2022, and further decreased to 85 in fifth grade in 2023. The cohort of students who were in second grade in 2019 initially consisted of 102 individuals, increased to 106 in fourth grade in 2021, remained at 106 in fifth grade in 2022, and reached 107 in sixth grade in 2023.

### 3.2. Changes in BMI and BMI Z-Scores Among All Students from 2019 to 2023 During the COVID-19 Lockdown: Cross-Sectional Study

**Figure 2:**
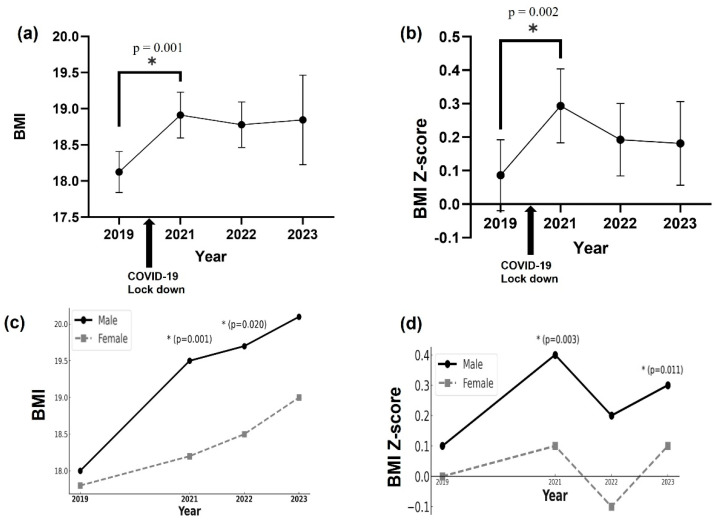
Cross-sectional changes in BMI and BMI Z-score by year for the overall student cohort (**a**,**b**) and by sex (**c**,**d**). * indicates statistically significant differences between groups or time points, as shown by the corresponding *p*-values.

The BMI and BMI Z-scores of all students increased significantly from 2019, 1 year before the COVID-19 lockdown, to 2021, 1 year after the lockdown (BMI: *p* = 0.001, Cohen’s d = 0.22; BMI Z-score: *p* = 0.002, Cohen’s d = 0.15) (Figure 2a,b). When analyzed by gender, male students showed a significant increase in both BMI and BMI Z-score from 2019 to 2021 (BMI: *p* = 0.001, Cohen’s d = 0.27; BMI Z-score: *p* = 0.008, Cohen’s d = 0.20). Female students did not show a statistically significant change, and the effect sizes were negligible (BMI: *p* = 0.109, Cohen’s d = 0.20; BMI Z-score: *p* = 0.182, Cohen’s d = −0.20) (Figure 2c,d). The effect sizes for BMI and BMI Z-score changes between 2019 and 2021 by sex and grade are detailed in Supplementary Table S1.

### 3.3. Changes in BMI and BMI Z-Scores of Students in Each Grade During the COVID-19 Lockdown: Longitudinal Study

#### 3.3.1. 2019 First-Grade Students: Longitudinal Analysis

**Figure 3:**
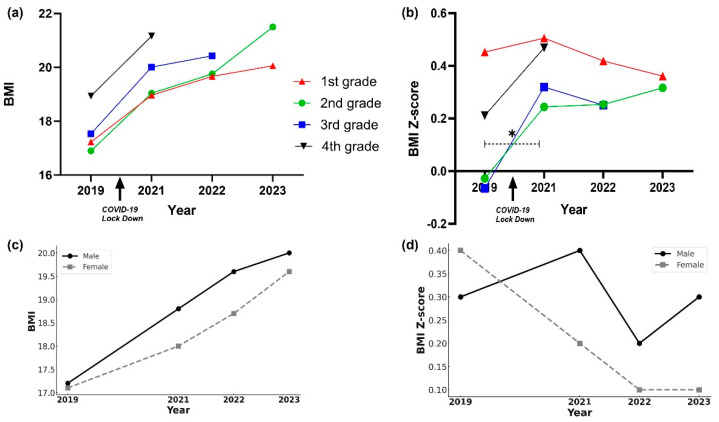
Change in BMI and BMI Z-score of students during the COVID-19 lockdown: longitudinal study: (**a**) BMI trajectories of elementary school students during a COVID-19 lockdown; (**b**) BMI Z-score trajectories of elementary school students during a COVID-19 lockdown; (**c**) BMI and (**d**) BMI Z-score from 2019 to 2023 in the 2019 first-grade cohort, by sex. * indicates statistically significant differences (*p* < 0.05) between pre- and post-lockdown periods, as detailed in the Results section.

When we followed the BMI of the students who were in first grade in 2019 until 2023, when they were in fifth grade, we observed that their average BMI increased with advancing grade levels (*p* < 0.001, Cohen’s d = 0.45) (Figure 3a, red line). However, the BMI Z-score showed no statistically significant difference from the first grade, before the COVID-19 lockdown, to the third grade, and then decreased significantly from the third to fifth grades post-lockdown (*p* = 0.003, Cohen’s d = 0.45) (Figure 3b, red line). Gender-specific analysis revealed that BMI (Figure 3c) increased in both male and female individuals with advancing grades (*p* < 0.001) but that the BMI Z-score (Figure 3d) decreased significantly only in female individuals from the first grade to the third and fourth grades (*p* = 0.025, Cohen’s d = −0.40).

#### 3.3.2. 2019 Second Grade Students: Longitudinal Analysis

In tracking the BMI of students who were in second grade in 2019 until they were in sixth grade in 2023, we observed that the overall BMI of the students increased significantly with each advancing grade (Figure 3a, green line) (*p* < 0.001, Cohen’s d = 0.35). The BMI Z-score increased significantly between the second and fourth grades, spanning the 2 years from before to after the COVID-19 lockdown (*p* < 0.001, Cohen’s d = 0.33) (Figure 3b, green line).

In the gender-specific analysis, we observed that BMI increased significantly for both male and female individuals with advancing grades (*p* < 0.001). However, the BMI Z-score increased significantly only in male individuals from the second to the fourth grade (*p* < 0.001, Cohen’s d = 0.38).

#### 3.3.3. 2019 Third-Grade Students: Longitudinal Analysis

When the cohort of students who were in third grade in 2019 was followed until they were in sixth grade in 2022, we note that their overall BMI increased significantly with each advancing grade (Figure 3a, blue line) (*p* < 0.001, Cohen’s d = 0.60). The BMI Z-score also increased significantly between the third and fifth grades, i.e., from pre- to post-COVID-19 lockdown (*p* < 0.001, Cohen’s d = 0.55) (Figure 3b, blue line). Gender-specific analysis revealed that both male and female individuals experienced significant increases in the BMI Z-score between the third and fifth grades (*p* < 0.001).

#### 3.3.4. 2019 Fourth-Grade Students: Longitudinal Analysis

In following the cohort of students who were in fourth grade in 2019 until they were in sixth grade in 2021, we observed that both their BMI (Figure 3a, black line) and BMI Z-score (Figure 3b, black line) showed significant increases overall and in gender-specific analyses from the fourth to the sixth grade (*p* < 0.001, Cohen’s d = 0.36 for BMI, 0.33 for BMI Z-score).

### 3.4. Difference in BMI and BMI Z-Score of Students Between 2019 and 2021: Longitudinal Study

**Figure 4:**
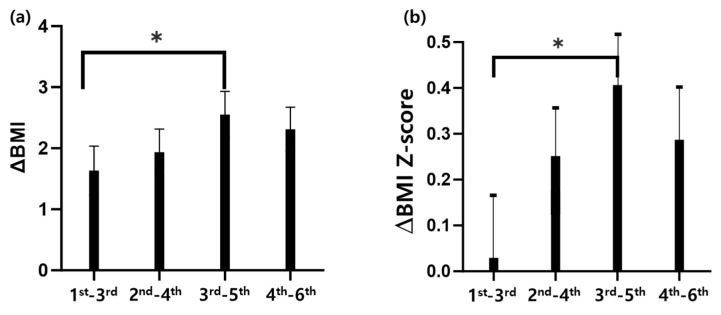
Impact of COVID-19 lockdown on BMI: a longitudinal study examining age-specific trends in elementary school children: (**a**) difference in BMI by grade level 2 years after COVID-19 lockdown; (**b**) difference in BMI Z-score by grade level 2 years after COVID-19 lockdown. * Significant difference between the groups based on the Tukey multiple comparison test.

By 2021, 2 years after the COVID-19 lockdown, the differences in BMI (ΔBMI) and in the BMI Z-score (ΔBMI Z-score) of children who were in first and second grades before the lockdown were smaller than those of children who were in the third and fourth grades in 2019. Notably, the students who were in third grade exhibited the most significant changes in BMI post-lockdown (Figure 4a,b).

### 3.5. Changes in BMI and BMI Z-Score of First and Second Graders According to Pre-COVID-19 Lockdown Obesity Status: Longitudinal Study

**Figure 5:**
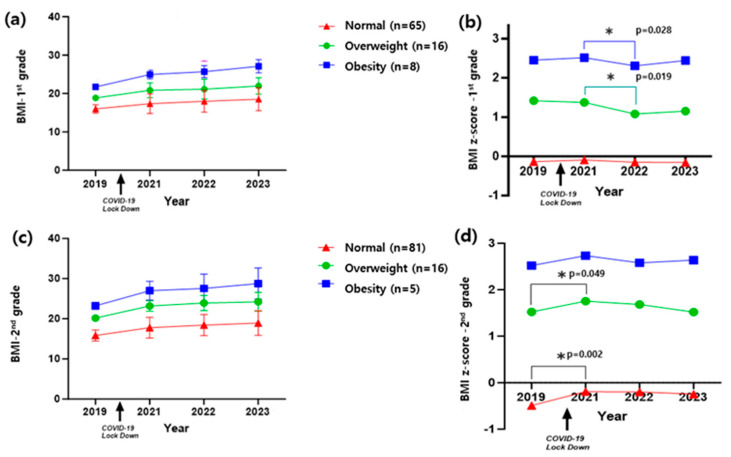
Longitudinal changes in BMI and BMI Z-score among 1st and 2nd graders stratified by pre-COVID-19 obesity status: (**a**) BMI trajectories in 1st graders by pre-lockdown obesity status; (**b**) BMI Z-score trajectories in 1st graders by pre-lockdown obesity status. (**c**) BMI trajectories in 2nd graders by pre-lockdown obesity status; (**d**) BMI Z-score trajectories in 2nd graders by pre-lockdown obesity status. * indicates a statistically significant difference in BMI Z-score between pre- and post-lockdown years (*p* < 0.05), as indicated by the adjacent *p*-values.

Among first graders, students classified as obese or overweight before the COVID-19 lockdown continued to show significant increases in BMI post-lockdown (Figure 5a). However, BMI Z-scores decreased significantly post-lockdown (Figure 5b) (*p* = 0.028 and *p* = 0.019). The normal BMI group did not show any notable differences from before to after the lockdown.

For second graders, BMI increased consistently, regardless of pre-lockdown obesity status (Figure 5c). Their BMI Z-scores showed significant increases from pre- to post-lockdown in the normal (*p* = 0.002) and overweight groups (*p* = 0.049) (Figure 5d).

## 4. Discussion

This study employed a retrospective longitudinal approach to evaluate how the COVID-19 lockdown impacted the BMI of elementary school students over a three-year span from 2019 to 2023, excluding the lockdown year, 2020. In cross-sectional analysis, a significant rise in BMI Z-scores was observed in 2021 compared to 2019, across both sexes. However, when analyzed longitudinally by academic grade, the results varied. First graders in 2019 showed a reduction in BMI Z-scores following the lockdown, whereas students who were in second to fourth grades experienced increases, most notably in the third-grade cohort.

However, longitudinal analysis by grade revealed contrasting effects: while children who were first graders in 2019 demonstrated a significant decrease in their BMI Z-score post-lockdown, those who were second, third, and fourth graders in 2019 showed a significant increase, which was most notable in third graders. Furthermore, we investigated pre-existing obesity status and found that students who were initially classified as obese or overweight in first grade continued to exhibit BMI increases post-lockdown, although their BMI Z-score decreased. This apparent paradox can be explained by the nature of the BMI Z-score, which reflects a child’s position relative to age- and sex-specific national growth standards. While BMI typically increases with age as part of normal growth, a decrease in BMI Z-score indicates that the child’s rate of BMI increase was slower than that of the reference population. This suggests a trend toward normalization, particularly among children who were overweight or obese prior to the lockdown. Taken together, the study findings highlighted the inconsistent and age-dependent effects of the COVID-19 lockdown on BMI.

These findings have several practical applications. First, they underscore the need for tailored interventions that consider age and pre-existing obesity levels to mitigate the long-term health consequences for children. Given the differing responses across grade levels, public health programs should prioritize age-specific strategies, potentially including targeted messaging about healthy eating and physical activity for different developmental stages. Second, our findings suggest that interventions focusing on younger children may benefit from increased parental involvement, while those for older children may need to emphasize independent decision-making skills and access to resources for healthy choices. These strategies can be implemented in schools, community centers, or primary care settings.

Our unexpected finding of decreased BMI in first graders after the COVID-19 lockdown, in contrast with the increases in older grades, necessitates further investigation. Several hypotheses, informed by existing literature [17,18,19], may offer explanations for this discrepancy. Increased parental involvement and oversight during lockdowns may have led to healthier diets and increased physical activity among younger children [13,20], a factor that may be less influential on older, more independent children. Changes in access to school meal programs, perhaps due to lockdown restrictions or remote learning, could have reduced access to higher-calorie options, contributing to weight loss in first graders. A shift toward structured home-based physical activity, which is more readily implemented with younger children, might also explain the difference. While seemingly paradoxical, given the overall BMI increase in other grades, increased parental stress and its potential impact on children’s appetite and eating habits [1] might also have played a role, although this would require further investigation within a broader context.

The observed rise in BMI among older elementary school students (grades 2–4) following the COVID-19 lockdown likely stemmed from a confluence of factors. Increased screen time [3,4,5,7] and reduced physical activity [3,4,5,6] due to school closures and activity restrictions contributed to a more sedentary lifestyle and decreased energy expenditure, which fosters weight gain [9,21,22]. Disrupted routines impacted dietary habits [3], potentially leading to irregular meal patterns and increased consumption of less healthy foods, exacerbated by stress and boredom. The lockdown also altered family dynamics [23] and mealtimes [24], potentially diminishing the emphasis on healthy eating. Furthermore, sleep disturbances [24,25] resulting from altered routines or heightened anxiety could have influenced hormone regulation and appetite, promoting weight gain. Limited access to regular healthcare during the lockdown [25] may have hindered early detection and management of weight-related problems. The more pronounced BMI increase in older children suggests that their greater autonomy in food choices and activities played a significant role. Socioeconomic factors also likely influenced access to healthy food and opportunities for physical activity [26]. Longitudinal studies are needed to ascertain the long-term consequences of these changes, as the observed increase may be temporary or may indicate lasting effects. A comprehensive understanding requires consideration of the interplay of these multiple factors and critical evaluation of the methodologies and limitations of individual studies.

While both the 3rd-to-5th-grade and 4th-to-6th-grade cohorts in this study were comprised of elementary school students, differences in developmental characteristics specific to upper elementary grades may have contributed to the observed divergence in ΔBMI Z-scores. First, the transition from 3rd to 5th grade often coincides with the onset of puberty, characterized by rapid growth and weight changes, potentially leading to a larger ΔBMI Z-score in this group. In contrast, students transitioning from 4th to 6th grade may be further along in puberty, or even post-pubertal, resulting in a deceleration of growth and a comparatively lower ΔBMI Z-score. Second, upper elementary students demonstrate increased capacity for self-regulation regarding diet and activity levels compared to their younger counterparts. Although the 3rd-to-5th-grade period marks an initial experience in independently managing dietary and exercise habits, the development of more mature self-regulatory skills by 4th-to-6th graders could lead to positive effects on weight management. Third, the influence of the home environment may also differ, with less parental oversight over dietary and activity choices for older children potentially dampening the impact.

Research on social lockdown has explored the impact thereof on the health of children and adolescents [1,27,28], particularly in relation to obesity, across various social contexts, even before the COVID-19 lockdown: studies had examined how lockdowns during natural disasters and societal crises affect the health of young people. For instance, research analyzing the health impacts of the lockdown following the 2011 Fukushima nuclear disaster in Japan [28,29,30,31,32,33] found a significant decrease in physical activity among adolescents, alongside the development of unbalanced eating habits, which led to increased obesity rates. This highlighted how large-scale societal disruptions due to disasters pose considerable challenges to maintaining healthy lifestyles.

Furthermore, earlier studies [28,34,35,36] that examined the effects of societal crises on child health demonstrated that factors such as illness, psychological stress, and social isolation elevate the risk of obesity among children and adolescents. Stress often manifests in poor eating habits, leading to emotional eating and subsequently contributing to obesity [14,37,38,39].

The global lockdowns due to the COVID-19 pandemic revealed similar patterns more clearly. Research indicated a marked reduction in physical activity during the lockdown period, with increased consumption of snacks and convenience foods at home [39]. Consequently, many children and adolescents experienced weight gain leading to rising obesity rates, as evidenced by various studies [14,37,38,39]. As lockdown durations persisted, the decline in sports and recreational activities exacerbated the obesity issue, which could have long-term detrimental effects on health [27]. Experts have emphasized the need for policy interventions to address changes in eating habits and physical activity during such lockdowns [1,8,40]. Such interventions are crucial for maintaining healthy lifestyles and implementing educational and support programs to prevent obesity.

The present study on BMI changes in elementary school children during and after the COVID-19 lockdown has several advantages over previous research but also has limitations. A major strength of this study was its longitudinal design, where the same children were tracked over multiple years, unlike previous studies that were mostly cross-sectional, providing only snapshots in time. Our approach allowed for a more accurate assessment of BMI changes that were directly attributable to the lockdown, minimizing confounding variables that can skew cross-sectional results. Another advantage of our study was the grade-specific analysis. Instead of treating all children as a homogenous group, we analyzed data by grade level, revealing age-specific variations in BMI response to the lockdown. This granular approach provides a more nuanced understanding than that provided by studies that used aggregated data. Further enhancing the analysis, our study incorporated pre-existing obesity status, allowing for a comparison of lockdown impacts on children already at risk and that on their normal-weight peers. This identified vulnerable populations and added depth to the interpretation of results. The use of both BMI and BMI Z-scores strengthened our analysis, providing a more robust assessment of weight status while accounting for age and sex differences.

In addition to statistical significance, we also evaluated the magnitude of changes using Cohen’s d effect size. The overall increase in BMI and BMI Z-scores between 2019 and 2021, although statistically significant, generally reflected small effect sizes (e.g., BMI: d = 0.22; BMI Z-score: d = 0.15). When stratified by sex, boys showed small to moderate effects (BMI: d = 0.27), while girls showed minimal or even negative changes in BMI Z-scores (d = −0.20). Notably, the 2019 third-grade cohort exhibited the largest increases in both BMI and BMI Z-score, with moderate effect sizes (d ≈ 0.4), suggesting that this developmental stage may be particularly sensitive to environmental disruptions such as pandemic-related restrictions. These findings highlight the importance of combining *p*-values with effect size interpretations to more accurately understand the clinical or public health implications of the observed trends.

Finally, several limitations of this study warrant cautious interpretation of the findings. Specifically, the focus on a single elementary school limits the generalizability of our findings to other populations or settings. The retrospective nature of the study, relying on pre-existing data, introduced potential biases related to data completeness and accuracy. While likely controlled for methodologically, the extracted data lacked explicit detail on how confounding variables (dietary changes, physical activity levels, and family stressors) were addressed.

Moreover, our analysis was restricted to anthropometric measurements (BMI and BMI Z-scores) collected through routine school health screenings. We were unable to assess other potentially influential factors, including dietary intake, physical activity, psychological stress, sleep patterns, genetic predisposition, and underlying endocrine or metabolic disorders. Additionally, individual COVID-19 infection status was not recorded, which may have influenced body composition or behavior during the lockdown period. These omissions limit our ability to fully interpret the causes behind BMI changes.

Furthermore, the lack of comprehensive body composition data (such as fat mass or muscle mass indices) and maturation status (e.g., pubertal staging) hindered a more refined understanding of the growth patterns observed. These physiological parameters may significantly influence BMI trajectories during late childhood. Finally, the exclusion of students with duplicate birthdays and the absence of longer-term follow-up data further limit the scope and duration of our conclusions. Future studies incorporating multidimensional health indicators and longer observation periods are essential to elucidate the full impact of pandemic-related disruptions on pediatric obesity.

## 5. Conclusions

This study revealed heterogeneous changes in BMI and BMI Z-scores among elementary school children following the COVID-19 lockdown. The impact varied depending on grade level, baseline obesity status, and sex, with third-grade students in 2019 exhibiting the most notable increases in both BMI and BMI Z-score. While BMI increased across all age groups, changes in BMI Z-scores were more nuanced, suggesting differential growth trajectories relative to standard growth patterns.

Importantly, although many of the observed changes were statistically significant, the corresponding Cohen’s d effect sizes were mostly small to moderate. This finding emphasizes that, beyond statistical significance, the magnitude of the observed changes must be interpreted with caution when assessing public health relevance.

While this study provides valuable insights into age- and risk group–specific vulnerability during pandemic-related disruptions, it does not establish causal relationships. Future studies incorporating behavioral, physiological, and psychosocial variables are needed to better understand the mechanisms behind these trends and to guide targeted interventions.

## Figures and Tables

**Table 1:** Longitudinal analysis of BMI and related demographic characteristics in elementary school students before and after the COVID-19 pandemic (2019–2023).

Year	Grade	Number	Ht	Ht-Zscore	Wt	Wt-Zscore	BMI	BMI-Zscore
2019	1	91	120.5 ± 5.4	0.1 ± 1.0	25.2 ± 5.4	0.3 ± 1.0	17.2 ± 2.5	0.4 ± 1.1
	2	102	127.0 ± 5.3	0.1 ± 0.9	27.4 ± 5.3	0.0 ± 1.0	16.8 ± 2.5	0.0 ± 1.1
	3	105	133.4 ± 5.6	0.1 ± 1.0	31.4 ± 6.9	0.0 ± 1.1	17.5 ± 3.0	0.0 ± 1.2
	4	76	139.3 ± 5.7	0.2 ± 0.9	37.1 ± 9.4	0.2 ± 1.4	18.9 ± 3.6	0.2 ± 1.6
	5	96	145.8 ± 6.8	0.1 ± 1.0	40.3 ± 10.6	0.0 ± 1.2	18.7 ± 3.6	−0.1 ± 1.2
	6	71	153.1 ± 7.8	0.3 ± 1.1	47.5 ± 10.7	0.2 ± 1.1	20.1 ± 3.8	0.1 ± 1.3
2021	1	75	122.2 ± 4.9	0.1 ± 1.0	25.5 ± 5.4	0.1 ± 1.2	16.8 ± 2.8	0.1 ± 1.4
	2	91	128.7 ± 5.3	0.2 ± 0.9	28.8 ± 6.0	0.2 ± 1.0	17.2 ± 2.7	0.0 ± 1.1
	3	90	134.4 ± 6.3	0.3 ± 1.0	34.6 ± 9.2	0.5 ± 1.2	18.9 ± 3.6	0.5 ± 1.3
	4	106	139.7 ± 6.0	0.2 ± 0.9	37.4 ± 8.5	0.2 ± 1.1	19.0 ± 3.5	0.2 ± 1.2
	5	105	146.7 ± 6.8	0.2 ± 1.0	43.4 ± 10.3	0.3 ± 1.2	20.0 ± 3.7	0.3 ± 1.2
	6	74	154.0 ± 6.5	0.4 ± 0.9	50.6 ± 12.3	0.5 ± 1.2	21.1 ± 4.1	0.4 ± 1.3
2022	1	78	122.5 ± 4.2	0.1 ± 0.8	24.3 ± 3.8	−0.0 ± 0.9	16.1 ± 2.1	−0.1 ± 1.1
	2	73	127.9 ± 5.2	0.2 ± 1.0	28.5 ± 6.4	0.1 ± 1.1	17.3 ± 3.0	0.1 ± 1.3
	3	91	134.9 ± 5.7	0.3 ± 0.9	33.5 ± 7.7	0.3 ± 1.1	18.2 ± 3.2	0.2 ± 1.2
	4	90	140.8 ± 6.9	0.2 ± 1.0	39.4 ± 11.0	0.4 ± 1.2	19.6 ± 3.9	0.4 ± 1.3
	5	106	146.6 ± 6.8	0.3 ± 1.0	42.8 ± 10.1	0.3 ± 1.1	19.7 ± 3.7	0.2 ± 1.3
	6	106	154.3 ± 6.7	0.5 ± 1.0	48.9 ± 11.2	0.4 ± 1.1	20.4 ± 3.8	0.2 ± 1.2
2023	1	82	122.5 ± 5.4	0.4 ± 0.9	25.2 ± 5.2	0.3 ± 1.1	16.6 ± 2.4	0.1 ± 1.2
	2	75	128.3 ± 4.6	0.3 ± 0.8	27.5 ± 4.8	0.0 ± 0.9	16.6 ± 2.3	−0.1 ± 1.1
	3	73	134.0 ± 5.4	0.2 ± 1.0	32.5 ± 7.8	0.1 ± 1.1	17.9 ± 3.4	0.0 ± 1.3
	4	91	139.8 ± 6.0	0.2 ± 0.9	37.5 ± 8.7	0.3 ± 1.1	19.0 ± 3.5	0.2 ± 1.2
	5	85	146.8 ± 7.0	0.4 ± 1.0	43.8 ± 12.2	0.4 ± 1.2	20.0 ± 4.0	0.3 ± 1.3
	6	107	152.9 ± 6.9	0.3 ± 1.0	50.2 ± 29.0	0.4 ± 1.5	21.4 ± 13.7	0.3 ± 1.9

Ht, Height; Wt, Weight; BMI, body mass index.

## Data Availability

The data supporting the findings of the study are available upon reasonable request from the corresponding author owing to privacy and ethical restrictions.

## Supplementary Materials

The following supporting information can be downloaded at https://www.mdpi.com/article/10.3390/children12091157/s1, Supplementary Table S1. Effect Sizes (Cohen’s d) for BMI and BMI Z-score by Grade Level (2019 vs. 2021).

## Abbreviations

The following abbreviations are used in this manuscript:

BMI	Body mass index
COVID-19	Coronavirus disease 2019
IRB	Institutional review board
