# Supplementary material for: Body Mass Index Changes from Before to 3 Years After the COVID-19 Lockdown: A Retrospective Longitudinal Study in a Single Elementary School
Source: Children (Basel). 2025 Aug 30;12(9):1157. doi: 10.3390/children12091157 (PMC12468548; doi:10.3390/children12091157)
Supplement: Supplementary file 1 [file children-12-01157-s001.zip › children-3651413-supplementary.pdf]

Supplementary Table S1. Effect Sizes (Cohen's d) for BMI and BMI Z-score by Grade Level (2019 vs. 2021)

Grade (2019)	Comparison Years	BMI Mean ± SD (Start)	BMI Mean ± SD (End)	Cohen's d (BMI)	BMI Z-score Mean ± SD (Start)	BMI Z-score Mean ± SD (End)	Cohen's d (Z-score)
1st	2019 vs. 2021	17.2 ± 2.5	16.8 ± 2.8	-0.15	0.4 ± 1.1	0.1 ± 1.4	-0.23
2nd	2019 vs. 2021	16.8 ± 2.5	17.2 ± 2.7	0.16	0.0 ± 1.1	0.0 ± 1.1	0.00
3rd	2019 vs. 2021	17.5 ± 3.0	18.9 ± 3.6	0.42	0.0 ± 1.2	0.5 ± 1.3	0.40
4th	2019 vs. 2021	18.9 ± 3.6	19.0 ± 3.5	0.03	0.2 ± 1.6	0.2 ± 1.2	0.00
5th	2019 vs. 2021	18.7 ± 3.6	20.0 ± 3.7	0.36	-0.1 ± 1.2	0.3 ± 1.2	0.33
6th	2019 vs. 2021	20.1 ± 3.8	21.1 ± 4.1	0.25	0.1 ± 1.3	0.4 ± 1.3	0.23
